# Development of a Machine Learning Model to Predict the Color of Extruded Thermoplastic Resins

**DOI:** 10.3390/polym16040481

**Published:** 2024-02-08

**Authors:** Puay Keong Neo, Yew Wei Leong, Moi Fuai Soon, Qing Sheng Goh, Supaphorn Thumsorn, Hiroshi Ito

**Affiliations:** 1 Graduate School of Organic Materials Science, Yamagata University, 4-3-16 Jonan, Yonezawa 992-8510, Yamagata, Japan; 2 Omni-Plus System Limited, 994 Bendemeer Road, 01-03 B-Central, Singapore 339943, Singapore; mfsoon.sg@ops-sys.com (M.F.S.); qsgoh.sg@ops-sys.com (Q.S.G.); 3 Matwerkz Technologies Pte Ltd., 994 Bendemeer Road, 01-03 B-Central, Singapore 339943, Singapore; enquiry@matwerkz.com; 4 Research Center for GREEN Materials and Advanced Processing, Yamagata University, 4-3-16 Jonan, Yonezawa 992-8510, Yamagata, Japan; thumsorn@yz.yamagata-u.ac.jp

**Keywords:** compounding, coloration, thermoplastic, machine learning, color prediction

## Abstract

The conventional method for the color-matching process involves the compounding of polymers with pigments and then preparing plaques by using injection molding before measuring the color by an offline spectrophotometer. If the color fails to meet the L*, a*, and b* standards, the color-matching process must be repeated. In this study, the aim is to develop a machine learning model that is capable of predicting offline color using data from inline color measurements, thereby significantly reducing the time that is required for the color-matching process. The inline color data were measured using an inline process spectrophotometer, while the offline color data were measured using a bench-top spectrophotometer. The results showed that the Bagging with Decision Tree Regression and Random Forest Regression can predict the offline color data with aggregated color differences (dE) of 10.87 and 10.75. Compared to other machine learning methods, Bagging with Decision Tree Regression and Random Forest Regression excel due to their robustness, ability to handle nonlinear relationships, and provision of insights into feature importance. This study offers valuable guidance for achieving Bagging with Decision Tree Regression and Random Forest Regression to correlate inline and offline color data, potentially reducing time and material waste in color matching. Furthermore, it facilitates timely corrections in the event of color discrepancies being observed via inline measurements.

## 1. Introduction

Color analysis stands as a crucial tool with a myriad of applications. Color analysis plays a pivotal role in determining tolerances for automotive coatings, ensuring the ultimate satisfaction of the end products. This becomes particularly crucial as automobiles are composed of a diverse range of materials. It is essential to verify that the color coating maintains a consistent and uniform appearance when applied to different materials with varying textures [1]. Additionally, Ariño et al. explored the impact of a plastic texture on color perception. Their conclusion highlighted the noteworthy influence that the texture of plastic exerts on color perception [2].

To create a standard for color communication during color analysis, the International Commission on Illumination (CIE) developed the CIE L* a* b* color space in 1976. The CIE 1976 L* a* b* color space is a three-dimensional, approximately uniform color space, produced by plotting in rectangular coordinates, L*, a*, and b* [3]. L* indicates lightness, a* is the red/green coordinate, and b* is the yellow/blue coordinate. The positive a* axis points roughly towards red color stimuli, the negative axis points approximately towards green stimuli, the positive b* axis points approximately towards yellow stimuli, and the negative b* axis points approximately towards blue stimuli. L* is associated with the luminance of the stimulus, making it a basic indicator of lightness [4]. The differences in L*, a *, and b* between two specimens, which are also referred to as Delta Values, are calculated using Equations (1)–(3).

(1) $$∆L^{*}=L_{Sample}^{*}-L_{Standard}^{*}$$

(2) $$∆a^{*}=a_{Sample}^{*}-a_{Standard}^{*}$$

(3) $$∆b^{*}=b_{Sample}^{*}-b_{Standard}^{*}$$

Historically, the measurement of color has typically been carried out via offline color measurements using offline bench-top spectrophotometers [5]. To achieve this, the materials must undergo molding into plates after the extrusion process. However, the preparation of samples for offline color measurement is a labor-intensive and time-consuming task, resulting in delayed measurement reports. This delay carries a significant risk of producing products that may not meet specifications during the waiting period [6].

An offline bench-top spectrophotometer serves as a specialized instrument, tailored for conducting color measurements and analyses in a laboratory or controlled environment. In contrast to inline spectrophotometers that are seamlessly integrated into production lines for real-time polymer melt flow measurements, the offline bench-top variant excels in delivering precise and accurate color measurements within a stationary setting.

The offline bench-top spectrophotometer incorporates a spherical interior. The design strategically obstructs the light source, directing it from the color chip and reflecting it at an 8-degree angle from the specimen. This configuration ensures that the reflected light is effectively captured by the detector, enabling precise and accurate color measurements. Two commonly employed measurement geometries in offline bench-top spectrophotometers are SCI and SCE [7].

Specular Component Included (SCI): In SCI measurements, the spectrophotometer captures all reflected light, including both specular and diffuse components. This effectively eliminates the impact of specular reflection from the surface, allowing the measurement to focus solely on color rather than appearance. As a result, SCI is universally adopted by companies for formulating color recipes.

Specular Component Excluded (SCE): In SCE measurements, the spectrophotometer selectively records only the diffuse reflection of light from the material’s surface, excluding specular reflection. This approach incorporates the surface appearance into the measurement. Consequently, SCE proves more valuable for quality control in the production process, especially when a balance between color and appearance is crucial.

**Figure 1:**
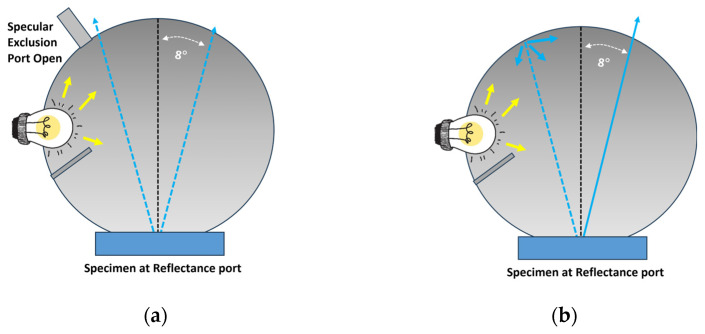
Working principle of an offline bench-top spectrophotometer with (**a**) Specular Component Excluded and (**b**) Specular Component Included.

Figure 1 shows the working principle of an offline bench-top spectrophotometer with (a) Specular Component Excluded geometry and (b) Specular Component Included geometry.

Conversely, an inline color measurement involves conducting direct color measurements on the polymer melt, which is already pigmented, preferably within the compound extruder itself [5], by using an inline process spectrophotometer (IPS). This allows operators to examine the polymer during production [8], and they are alerted as soon as the color begins to deviate out-of-spec so that corrections can be made immediately to minimize product rejects and wastage.

**Figure 2:**
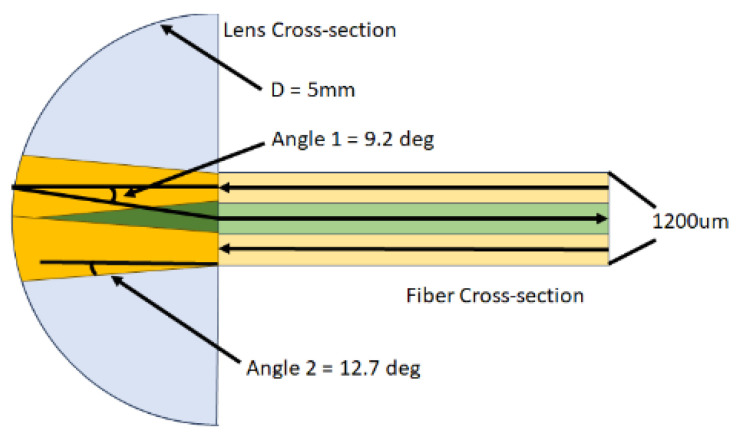
Working principle of inline process spectrophotometer.

The IPS works by illuminating the molten polymer within the die using light from the source at Angle 2, which travels through the fiber optics and a Reflection Polymer Melt Probe (RPMP). The reflected signal from the polymer melt is then captured at Angle 1 and transported back to the IPS. [9] Angle 1 aims to closely approximate the sphere measurement (commonly referred to as diffuse/8°) of a bench-top spectrophotometer, which is set at 8 degrees. However, owing to equipment constraints, the optimal angle that it can attain is Angle 1. Figure 2 shows the working principle of the inline process spectrophotometer.

However, in previous research, it was observed that the scale of the colors of inline and offline color measurements are distinctly different [10]. Specifically, the color change in inline measurements is minimal, whereas it registers as significantly more pronounced in offline measurements. This discrepancy emphasizes the need for refined approaches in handling data from these two distinct measurement methods. Addressing this incongruity will facilitate the prediction of the CIE L*, a*, and b* values for the output solid polymer based on the inline color measurement, enabling corrections in case of any detected deviations and averting the rejection of the entire production batch.

In recent times, there has been a discernible shift towards the application of machine learning algorithms and artificial intelligence to model and optimize the relationship between input and output variables. Illustrating this trend is Lee’s study, where an Artificial Neural Network (ANN) was implemented. The ANN was specifically designed to predict product properties such as mass, diameter, and height [11]. Shams-Nateri’s study also demonstrated an application of Neural Networks to relate the color of fibers in the mentioned directions [12]. Jeon constructed machine learning models to predict the melting temperature after plasticization [13]. Joo devised three models to predict the physical properties of PP composites, employing three distinct machine learning (ML) methods: Multiple Linear Regression (MLR), Deep Neural Network (DNN), and Random Forest (RF) [14].

The utilization of machine learning algorithms to develop predictive models from training data demonstrates significant potential for enhancing product quality and minimizing waste and downtime in the polymer processing industries [15]. However, a common drawback that has been observed in many of the machine learning approaches and highlighted in the literature is the opaque nature of these algorithms. Often, it becomes challenging to discern the reasons behind the model’s accurate predictions, as they provide little insight into the underlying process factors and relationships influencing the output [16].

The objective of this study is to design a machine learning model to predict the offline color measurement data using the inline color measurement and material dosage as input parameters. To achieve this, Bagging with Decision Tree Regression, Deep Neural Network, Multiple Linear Regression, and Random Forest Regression are used as the machine learning model. The performance of the model will be evaluated using aggregated dE, which is similar to the root mean square error (RMSE). The insights gained from this study will facilitate the real-time monitoring and prediction of offline color data during compounding through the utilization of inline color data. This approach enables timely corrections to be implemented in the event of any detected deviations.

## 2. Materials and Methods

### 2.1. Materials

In this study, a compounding process, followed by an injection molding process, was conducted to gather a well-diversified set of data for training the machine learning models. The materials employed in this study include polycarbonate resin, dispersing agent, and pigments. Polycarbonate (PC) (Makrolon^®^ 2807) was supplied by Covestro, Singapore. PC Makrolon 2807 has a density of 1.20 g/cm^3^ and a melt flow rate (MFR) of 10 g/10 min (measured at 300 °C/1.2 kg). Polycarbonate was chosen for its high usage in engineering plastic manufacturing. Ethylene Bis Stearamide (EBS) L-205F dispersing agent was supplied by DP Chemicals Pte Ltd., Singapore. Pigments, which included Tiona 288, Raven 1010, Heliogen Green K8730, Ultramarine Blue 05, Solvent Yellow 114, and Plast Red 8355, were supplied by Hexachem (M) Sdn. Bhd, Selangor, Malaysia, and DP Chemicals Pte Ltd., Singapore.

Formulations crafted for PC experiments are tabulated in Table 1. The components in each formulation were first manually hand-tumbled to ensure uniformity before feeding them into the extruder.

### 2.2. Compounding Equipment

**Figure 3:**
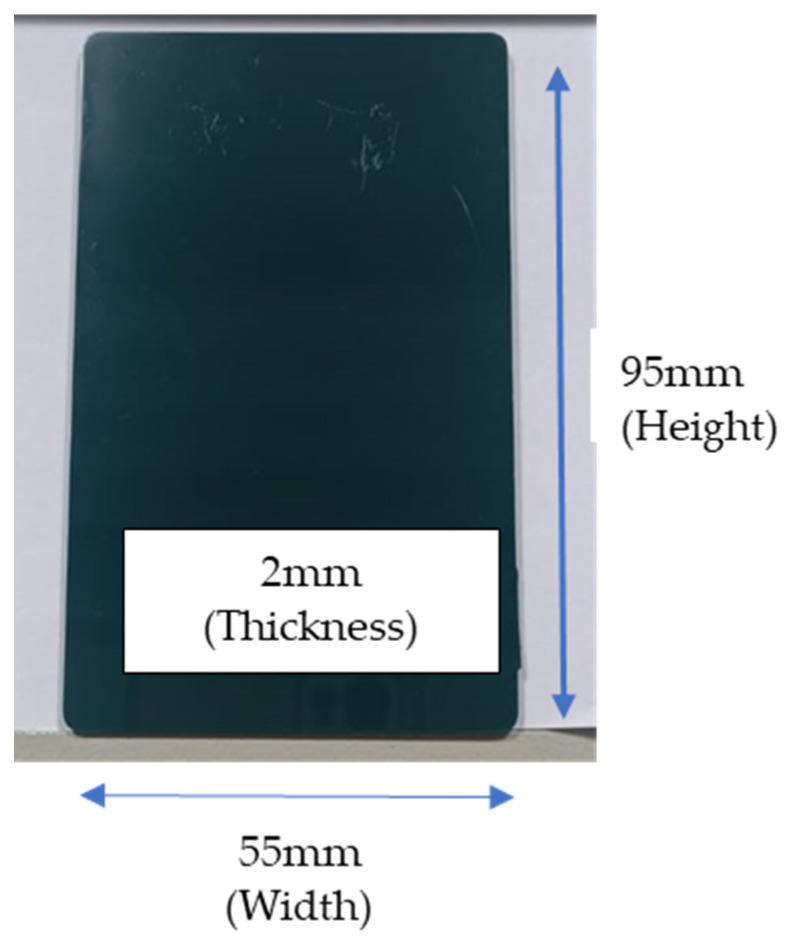
Image of injection-molded color chip used in this study.

Compounding was performed by using an intermeshing co-rotating twin screw extruder (Coperion GmbH, Stuttgart, Germany). It has a 26 mm screw diameter, an L-to-D ratio of 44, is powered by a 27-kW motor, and features 11 heating zones for the barrel along with one for the die. The barrel temperatures were set at 260–280 °C for PC, with a screw speed of 230 rpm. Upon exiting the die, the extrudate was quenched in cold water, dried using air, and then converted into pellets via a pelletizer. The pellets were then molded via injection molding (Sumitomo C250, Singapore) with a clamp tonnage of 100 tons into a cuboid color chip (95 mm by 55 mm by 2 mm), as shown in Figure 3. The dimension of the color chip was selected based on the industrial standard in the polymer compounding industry. The injection barrel temperature was set at 260–280 °C at an injection speed of 120 mm/s, with mold temperatures of 100 °C. The specimen was conditioned at 23 ± 2 °C for 24 h before color measurements.

### 2.3. Color Measurement

In our experiment, the color measurement of the polymer melt was conducted using Equitech’s EQUISPEC™ Inline Process Spectrophotometer (IPS) (Equitech, Charlotte, NC, USA), along with a Reflection Polymer Melt Probe (RPMP). The RPMP was mounted at the die head of the extruder and ensured that there was ample shear force to consistently cause the new polymer melt to shear across the RPMP.

The data acquisition rate of the color measurement of polymer melts was set at every 2 s. The CIE L* a* b* color reading [3] from the spectrophotometer was recorded as the inline measurement by using D65 as the standard illuminant [17] and a standard observer angle of 10 degrees. IPS has a measurement uncertainty of 0.01 unit for CIE L* a* b* color reading. The mean data were only collected after 5 min when the reading is stabilized and shown in Table A1. The measurement period was 5 min.

For offline color measurement, we used an X-Rite Ci7800 bench-top Spectrophotometer [18] (X-Rite—Southeast Asia and Pacific, Singapore) with a 400 mm UV filter, equipped with Color iMatch professional software (Version 10.7.2). A 10° supplementary standard observer and D65 illuminant [17] were used, coupled with SCI mode. Given that the surface texture can induce diffusion and scattering of light, influencing color appearance [19], the SCI mode was exclusively preferred for assessing color rather than appearance. The CIE L* a* b* color readings from the spectrophotometer were documented as offline measurements. The bench-top spectrophotometer used in this study has a measurement uncertainty of 0.01 unit for CIE L* a* b*. The mean data were calculated based on the average reading of 10 pieces of color chips for each dosage and shown in Table A1.

## 3. Machine Learning Architectures

In this paper, four machine learning models were employed for predictions: Bagging with Decision Tree Regression, Deep Neural Network, Multiple Linear Regression, and Random Forest Regression.

### 3.1. Bagging with Decision Tree Regression

The Bagging with Decision Tree Regression model is a combination of Bagging Regression and Decision Tree Regression.

**Figure 4:**
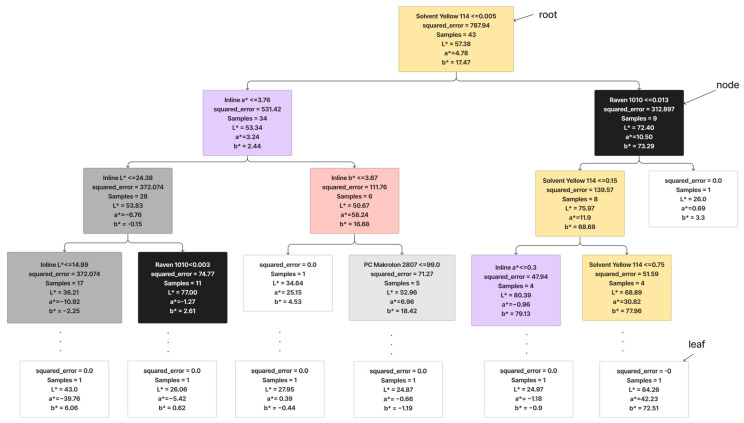
Working principle of decision trees.

A Decision Tree Regression is a predictive model that maps features of an input to make decisions or predictions [20]. In the context of regression, it is used to predict a continuous outcome based on input features [21]. The tree structure consists of nodes representing decisions based on features and leaves representing the predicted outcomes [22]. Figure 4 shows an example of the Decision Tree Regression that was generated in this study. The decision tree starts with a root condition of Solvent Yellow 114 with a dosage under 0.005, where it best splits the data to minimize the mean squared error (MSE). It then creates a condition for splitting the data, aiming to reduce the variance in the predicted values which is inline a* ≤ 3.78 and Raven 1010 ≤ 0.013. The recursive splitting process continues, forming a binary tree structure. The goal is to iteratively partition the data into subsets that exhibit lower variance in the target variable. As the tree grows, leaf nodes contain the predicted values for the target variable, which might be the mean or median of the target values in the leaf. During the prediction phase, a new data point traverses the tree, following the path of decisions until it reaches a leaf node. The predicted value is then determined by the value that is associated with that leaf.

**Figure 5:**
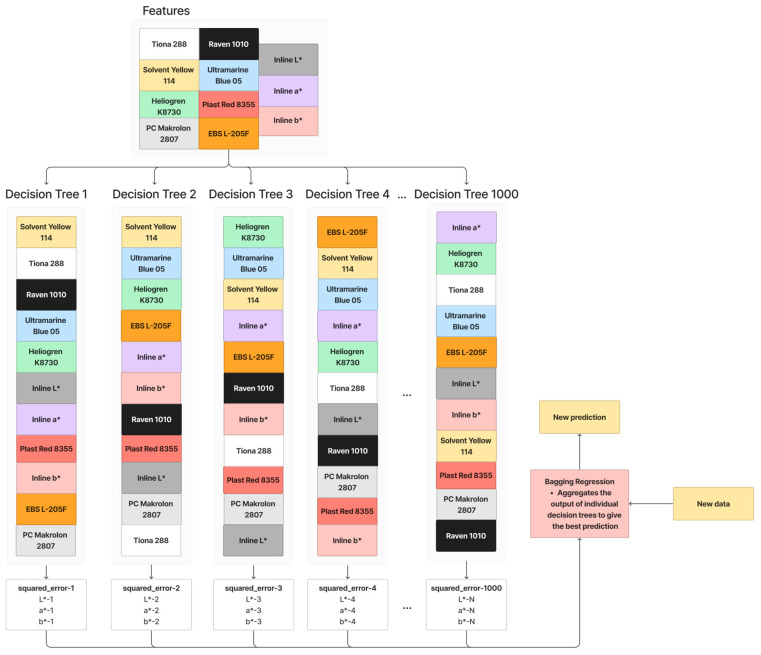
Working principle of Bagging with Decision Tree Regression.

Bagging Regression is an ensemble learning technique that involves training multiple decision trees with different feature orders [23]. Figure 5 shows the working principle of Bagging Regression. In this process, features are randomly selected and arranged to create decision trees. This is repeated multiple times (1000 times in this paper), resulting in a collection of diverse decision trees. When making predictions for new data, the Bagging Regression aggregates the outputs of these individual trees, often by averaging, to provide a more robust and generalized prediction. The randomness introduced in feature selection and ordering helps reduce overfitting, making the model more effective and resilient. The parameters for Bagging with Decision Tree Regression used in this paper are summarized in Table 2.

### 3.2. Deep Neural Network

**Figure 6:**
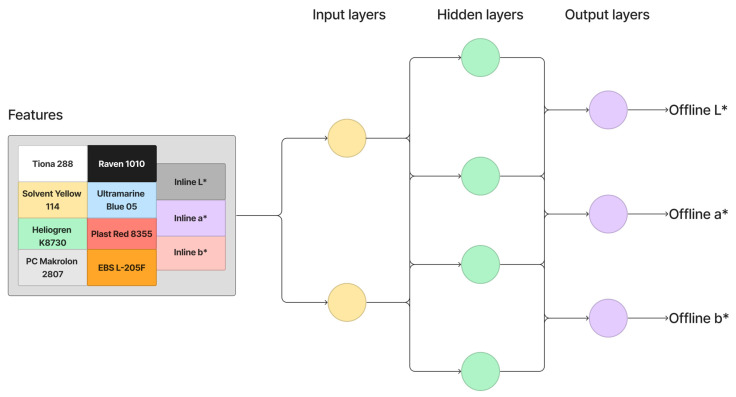
Working principle of Deep Neural Networks.

A Deep Neural Network makes predictions through a process called forward propagation, which involves passing the input data through the network’s layers of interconnected neurons. Figure 6 shows the working principle of the Deep Neural Network. In this paper, the network is trained for 50 epochs, with each epoch processing batches of 32 samples at a time, considering the small sample size. The Deep Neural Network comprises three layers: an input layer with 128 neurons using Rectified Linear Unit (ReLU) activation, a hidden layer with 64 neurons and ReLU activation, and an output layer with three neurons corresponding to the targets (offline L*, a*, b*). The network is compiled using the Adam optimizer and the mean squared error loss function, commonly chosen for regression problems. Once trained, the Deep Neural Network is utilized to make predictions on new dataset features. The parameters for the Deep Neural Network in this paper are summarized in Table 3.

### 3.3. Multiple Linear Regression

Multiple Linear Regression makes predictions by combining the weighted sum of multiple input features with a constant term. In our paper, the input features are the material dosage and inline L* a* b*. The model learns these weights during training to minimize the difference between its predictions and the actual target values, allowing it to generalize and make accurate predictions on new data by considering multiple input features simultaneously. The parameters for Multiple Linear Regression in this paper are summarized in Table 4.

### 3.4. Random Forest Regression

**Figure 7:**
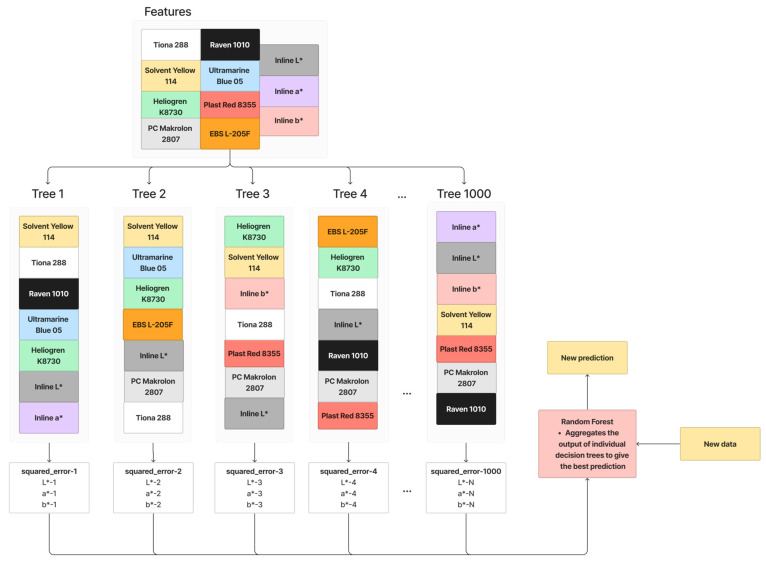
Working principle of Random Forest.

Random Forest Regression, an ensemble learning technique, refines Bagging principles by introducing more randomization into the construction of individual decision trees. In contrast to Bagging with a Decision Tree Regression, Random Forest Regression selects only a random subset of features, not all, for splitting a node. This deliberate feature subset randomness aims to decrease correlations between trees, enhancing the overall robustness. However, it may potentially miss crucial features. The working principle of Random Forest Regression is illustrated in Figure 7, where a subset of features is randomly chosen for each tree’s training. This process is iterated 1000 times. When given new data, the Random Forest Regression aggregates outputs from individual trees to provide a more robust prediction. The parameters for Random Forest Regression in this paper are summarized in Table 5.

## 4. Machine Learning Methodology

### 4.1. Data Exploration through the Pearson Correlation Coefficient

To enhance prediction accuracy, understanding the linear relationship between features—material dosage and inline color data and the target variables—and offline color data is crucial. The Pearson correlation coefficient (PCC) was employed for this purpose. The PCC measures the strength and direction of the linear relationship between two variables. The PCC was computed using Equation (4).

(4) $$r=\frac{\sum (X_{i}-\bar{X})(Y_{i}-\bar{Y})}{\sqrt{{\sum (X_{i}-\bar{X})}^{2}\cdot {\sum \left(Y_{i}-\bar{Y}\right)}^{2}}}$$

where *X_i_* and *Y_i_* are individual data points of the variables *X* and *Y*, and $\bar{X}$ and $\bar{Y}$ are the means of variables *X* and *Y*, respectively.

This analysis was primarily undertaken to identify any linear relationships and the necessity of data augmentation for improved model performance. Moreover, the statistical significance of these correlations was assessed to ensure that the observed relationship is not due to random chance but reflects a genuine association in the data.

Table 6 illustrates the correlations between each material and the offline a* value. It is evident that materials that are strongly correlated with the offline a* value include the red pigment, indicating a positive linear relationship, and the blue pigment, indicating a negative linear relationship. This discovery piques interest, given that the a* value is typically impacted by the dosage of both red and green pigments. Nevertheless, the difference in correlation coefficients between green and blue pigments is not considerable. Therefore, it is reasonable to propose that the blue pigment tends to exhibit a greenish tone.

Table 7 illustrates the correlations between each material and the offline b* value. The yellow pigment (revealing a positive linear relationship) and blue pigment (revealing a negative linear relationship) exhibit strong correlations with the b* value. This aligns with the CIE L* a* b* color space, where the yellow pigment contributes a positive b* value, and the blue pigment contributes a negative b* value.

Table 8 presents the correlations between each material and the offline L* value. The results show that the white pigment (indicating a positive linear relationship), as well as the blue and black pigments (indicating a negative linear relationship), demonstrate strong correlations with the L* value. The positive association with the L* value aligns with the CIE L* a* b* color space, where the white pigment contributes to a positive L* value.

It is noteworthy that, apart from the black pigment, the blue pigment also significantly contributes to the negative L* value. This observation suggests that the blue pigment could serve as an alternative to the black pigment in contributing to a negative L* value.

Table 9 displays the correlations between the inline and offline color data, revealing robust associations between the two. The results show that strong correlations between inline and offline color data affirm the potential of inline color data to predict offline color characteristics. These correlations between material dosage, inline L* a* b*, and offline L* a* b* values are critical for identifying relevant features in model training, guiding the approach towards more precise and reliable color prediction.

In summary, these findings not only demonstrate significant linear relationships between the chemical components and color data but also adhere to the established principles of the CIE L* a* b* color space. This enhances the understanding of how different materials influence color properties, which is vital for developing more accurate machine learning models for color prediction.

### 4.2. Dataset Allocation

Out of the complete dataset that was generated from the color measurement, which comprises 83 color formulations as presented in Table 1, 74 datasets were designated for training, and the remaining 9 datasets were for testing to assess model performance. The order of the data was randomized before splitting to reduce overfitting and improve the generalization of the data.

**Figure 8:**
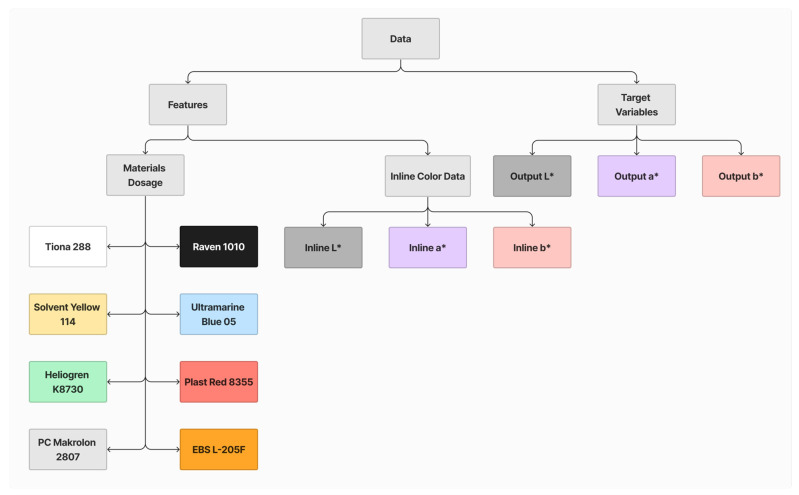
Summary of features and target variables.

Each dataset comprises 11 features and 3 target variables, as illustrated in Figure 8. The features are categorized into two groups: the dosage of each material and inline L* a* b*. The target variables include offline L* a* b* values. These datasets will be employed for model training and performance evaluation.

### 4.3. Evaluation Metric

The performance of each model was assessed using the aggregated dE, a domain-specific RMSE. RMSE is defined as the standard metric in regression analysis that measures the average magnitude of the errors between predicted and actual values. An aggregated dE gauges the average color difference between predicted and actual values of the test dataset, calculated using Equation (5). Equation (6) shows the equation for calculating RMSE. Lower dE values signify greater accuracy in the model prediction.

(5) $$Aggregated\;dE^{*}=\sqrt{\sum_{i=1}^{n}\frac{{{(\hat{L}}_{i}^{*}-L_{i}^{*})}^{2}+{{(\hat{a}}_{i}^{*}-a_{i}^{*})}^{2}+{{(\hat{b}}_{i}^{*}-b_{i}^{*})}^{2}}{n}}$$

${\hat{L}}_{1}^{*}$, ${\hat{L}}_{2}^{*}$,…, ${\hat{L}}_{n}^{*}$, are predicted L* values, and $L_{1}^{*}$, $L_{2}^{*}$,… $L_{n}^{*}$ are actual L* values.

${\hat{a}}_{1}^{*}$, ${\hat{a}}_{2}^{*}$,…, $a_{n}^{*}$, are predicted a* values, and $a_{1}^{*}$, $a_{2}^{*}$,… a are actual a* values.

${\hat{b}}_{1}^{*}$, ${\hat{b}}_{2}^{*}$,…, ${\hat{b}}_{n}^{*}$, are predicted b* values, and $b_{1}^{*}$, $b_{2}^{*}$,… $b_{n}^{*}$ are actual b* values.

n is the number of samples.

(6) $$RMSE=\sqrt{\sum_{i=1}^{n}\frac{{{(\hat{y}}_{i}^{*}-y_{i}^{*})}^{2}}{n}}$$

${\hat{y}}_{1}^{*}$, ${\hat{y}}_{2}^{*}$,…, ${\hat{y}}_{n}^{*}$, are predicted values, and $y_{1}^{*}$, $y_{2}^{*}$,… $y_{n}^{*}$ are actual values.

n is the number of samples.

## 5. Results

### 5.1. Performance of Machine Learning Model

Table 10 displays the aggregated dE of each model at a sample size of 83 and a test sample size of 9. Both Bagging with Decision Tree Regression and Random Forest exhibit the lowest aggregated dE values of 10.84 and 10.75, respectively. In contrast, the Deep Neural Network demonstrates a higher aggregated dE, indicating overfitting caused by the limited sample size. Multiple Linear Regression also exhibits a high aggregated dE due to its inability to capture complex, nonlinear relationships that are present in the dataset, limiting its predictive accuracy.

In summary, Bagging with Decision Tree Regression and Random Forest Regression exhibit the lowest aggregated dE values. However, the observed color difference remains too high for practical production use. The impact of the sample size on reducing the color difference will be explored in the next section to assess the feasibility of achieving more satisfactory results.

### 5.2. Impact of Sample Size on Machine Learning Accuracy

**Figure 9:**
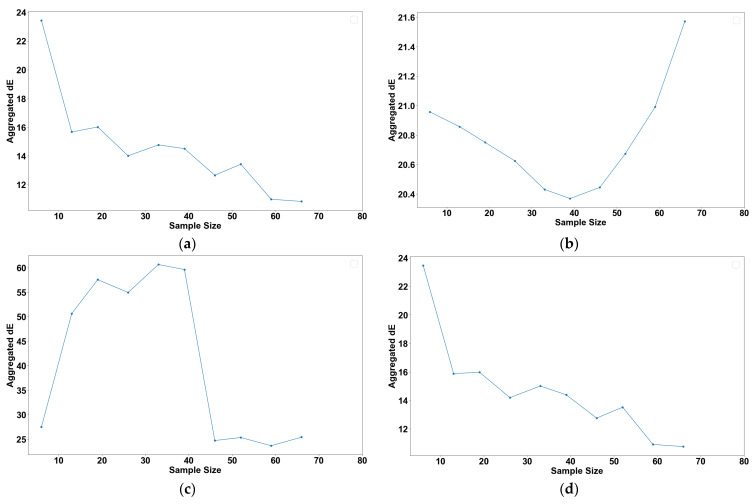
Plot of root mean square error against sample size for (**a**) Bagging with Decision Tree Regression, (**b**) Deep Neural Network, (**c**) Multiple Linear Regression, and (**d**) Random Forest Regression.

To understand the effect of the sample size on model accuracy, a systematic analysis of how increasing sample sizes influence the aggregated dE for various models that are referenced in this paper was conducted. Each model architecture was trained and evaluated using various sample sizes. These samples were obtained as random subsets of the total training samples and selected without replacement, ensuring the uniqueness and variability of each sample set. Figure 9 illustrates the aggregated dE plotted against the number of samples for each model type.

In Figure 9a, a decline in the aggregated dE is observed for Bagging with Decision Tree Regression as the sample size increases. This trend suggests enhanced predictive accuracy, likely due to the model’s exposure to a broader range of feature variations within the larger datasets.

Conversely, Figure 9b demonstrates a decrease in the aggregated dE for the Deep Neural Network model up to a sample size of 45. Beyond this point, the aggregated dE increases. There might be several explanations for this, but it is likely an overfitting issue, as the model learns the noise of the additional data instead of capturing the underlying patterns [24].

Figure 9c reveals an initial rise in the aggregated dE for Multiple Linear Regression with increasing sample sizes, followed by a decrease after reaching 45 samples. This pattern is consistent with findings in Knofczynski’s research, underscoring the necessity of a minimum sample size for accurate predictions [25]. Smaller sample sizes might result in misleading outcomes due to insufficient data representation.

Finally, Figure 9d shows that the Random Forest Regression exhibits a trend that is akin to Bagging with Decision Tree Regression. The aggregated dE decreases as more samples are introduced, which is expected given their common underlying mechanism based on Decision Tree Regression.

These observations suggest that the Bagging with Decision Tree Regression and Random Forest Regression provide the highest and most consistent returns (in terms of aggregated dE) for a given increase in the dataset compared to other models.

Table 11 highlights the pros and cons of our study compared with previous studies.

## 6. Conclusions

In this study, machine learning algorithms were developed to predict offline color data using both inline color measurements during polymer melt compounding and offline color measurements on injection-molded cuboid color chips. Four machine learning models, namely, Bagging with Decision Tree Regression, Deep Neural Network, Multiple Linear Regression, and Random Forest Regression, were employed with the input of measurement data and material dosage.

Among these models, Bagging with Decision Tree Regression and Random Forest Regression demonstrated notable effectiveness, achieving the lowest aggregated dE values of 10.84 and 10.75. As the current aggregated dE values are somewhat high for production-level application, further analysis of the effect of the sample on model prediction accuracy is required. Bagging with Decision Tree Regression and Random Forest Regression show a consistent reduction in aggregated dE values with an increasing sample size. This suggests that the truth function for offline color is easily discoverable by increasing the training sample size.

This methodology suggests a potentially more efficient approach to ensure color chip conformity during production. As the model performance improves with the training dataset size, the minimization of material and time wastage becomes more achievable. Overall, the results indicate a promising avenue for integrating machine learning into color quality control processes within the polymer manufacturing industry.

## Figures and Tables

**Table 1:** Formulation of polycarbonate with different pigments to build the dataset.

Formulation	PC Makrolon 2807	EBS L-205F	Tiona 288	Raven 1010	Heliogen Green K 8730	Ultramarine Blue 05	Solvent Yellow 114	Plast Red 8355
1	100	0	0	0	0	0	0	0
2	99.65	0.3	0.05	0	0	0	0	0
3	99.6	0.3	0.1	0	0	0	0	0
4	99.45	0.3	0.25	0	0	0	0	0
5	99.2	0.3	0.5	0	0	0	0	0
6	98.7	0.3	1	0	0	0	0	0
7	97.7	0.3	2	0	0	0	0	0
8	96.7	0.3	3	0	0	0	0	0
9	94.7	0.3	5	0	0	0	0	0
10	98.7	0.3	0.999	0.001	0	0	0	0
11	98.7	0.3	0.995	0.005	0	0	0	0
12	98.7	0.3	0.99	0.01	0	0	0	0
13	98.7	0.3	0.96	0.04	0	0	0	0
14	98.7	0.3	0.7	0.3	0	0	0	0
15	98.7	0.3	0.5	0.5	0	0	0	0
16	99.69	0.3	0	0	0.01	0	0	0
17	99.68	0.3	0	0	0.02	0	0	0
18	99.65	0.3	0	0	0.05	0	0	0
19	99.6	0.3	0	0	0.1	0	0	0
20	99.5	0.3	0	0	0.2	0	0	0
21	99.3	0.3	0	0	0.4	0	0	0
22	99.1	0.3	0	0	0.6	0	0	0
23	98.7	0.3	0	0	1	0	0	0
24	99.69	0.3	0	0.01	0	0	0	0
25	99.68	0.3	0	0.02	0	0	0	0
26	99.65	0.3	0	0.05	0	0	0	0
27	99.6	0.3	0	0.1	0	0	0	0
28	99.5	0.3	0	0.2	0	0	0	0
29	99.3	0.3	0	0.4	0	0	0	0
30	99.1	0.3	0	0.6	0	0	0	0
31	98.7	0.3	0	1	0	0	0	0
32	99.69	0.3	0	0	0	0.01	0	0
33	99.68	0.3	0	0	0	0.02	0	0
34	99.65	0.3	0	0	0	0.05	0	0
35	99.6	0.3	0	0	0	0.1	0	0
36	99.5	0.3	0	0	0	0.2	0	0
37	99.3	0.3	0	0	0	0.4	0	0
38	99.1	0.3	0	0	0	0.6	0	0
39	98.7	0.3	0	0	0	1	0	0
40	99.69	0.3	0	0	0	0	0.01	0
41	99.68	0.3	0	0	0	0	0.02	0
42	99.65	0.3	0	0	0	0	0.05	0
43	99.6	0.3	0	0	0	0	0.1	0
44	99.5	0.3	0	0	0	0	0.2	0
45	99.3	0.3	0	0	0	0	0.4	0
46	99.1	0.3	0	0	0	0	0.6	0
47	98.7	0.3	0	0	0	0	1	0
48	98.7	0.3	0.95	0	0	0	0.05	0
49	98.7	0.3	0.9	0	0	0	0.1	0
50	98.7	0.3	0.7	0	0	0	0.3	0
51	98.7	0.3	0.5	0	0	0	0.5	0
52	98.7	0.3	0.48	0.02	0	0	0.5	0
53	98.7	0.3	0	0.025	0	0	0.975	0
54	98.7	0.3	0	0.05	0	0	0.95	0
55	98.7	0.3	0.95	0	0	0.05	0	0
56	98.7	0.3	0.9	0	0	0.1	0	0
57	98.7	0.3	0.7	0	0	0.3	0	0
58	98.7	0.3	0.5	0	0	0.5	0	0
59	98.7	0.3	0.48	0.02	0	0.5	0	0
60	98.7	0.3	0	0.025	0	0.975	0	0
61	98.7	0.3	0	0.05	0	0.95	0	0
62	98.7	0.3	0.95	0	0	0.05	0	0
63	98.7	0.3	0.9	0	0	0.1	0	0
64	98.7	0.3	0.7	0	0	0.3	0	0
65	98.7	0.3	0.5	0	0	0.5	0	0
66	98.7	0.3	0.48	0.02	0	0.5	0	0
67	98.7	0.3	0	0.025	0	0.975	0	0
68	98.7	0.3	0	0.05	0	0.95	0	0
69	99.69	0.3	0	0	0	0	0	0.01
70	99.68	0.3	0	0	0	0	0	0.02
71	99.65	0.3	0	0	0	0	0	0.05
72	99.6	0.3	0	0	0	0	0	0.1
73	99.5	0.3	0	0	0	0	0	0.2
74	99.3	0.3	0	0	0	0	0	0.4
75	99.1	0.3	0	0	0	0	0	0.6
76	98.7	0.3	0	0	0	0	0	1
77	98.7	0.3	0.95	0	0	0	0	0.05
78	98.7	0.3	0.9	0	0	0	0	0.1
79	98.7	0.3	0.7	0	0	0	0	0.3
80	98.7	0.3	0.5	0	0	0	0	0.5
81	98.7	0.3	0.48	0.02	0	0	0	0.5
82	98.7	0.3	0	0.025	0	0	0	0.975
83	98.7	0.3	0	0.05	0	0	0	0.95

**Table 2:** Machine learning model architecture of Bagging with Decision Tree Regression.

Parameter	Value
Random state for Decision Tree Regression	42
Number of base estimators	1000
Random state for Bagging Regression	42
Number of parallel jobs	−1

**Table 3:** Machine learning model architecture of Deep Neural Networks.

Parameter	Value
Number of hidden layers	2
Number of input layer neurons	11
Number of hidden layer neurons	192
Number of output layer neurons	3
Hidden layer activation function	Relu
Optimizer	Adam
Loss function	RMSE
Training iterations (epochs)	50
Batch size	32

**Table 4:** Machine learning model architecture of Multiple Linear Regression.

Parameter	Value
fit_intercept	True
Normalize	False
Copy_X	True
Number of jobs	None

**Table 5:** Machine learning model architecture of Random Forest.

Parameter	Value
Random state for Random Forest	42
Number of base estimators	1000

**Table 6:** Pearson correlation coefficients between chemical components and offline a* value.

Chemical Component	Offline Color Data	Pearson Correlation Coefficient
Plast Red 8355	Offline a* value	0.355185
Solvent Yellow 114	Offline a* value	0.119745
EBS L-205F	Offline a* value	0.021655
PC Makrolon 2807	Offline a* value	0.001232
Raven 1010	Offline a* value	−0.048207
Tiona 288	Offline a* value	−0.074003
Heliogen Green K 8730	Offline a* value	−0.097658
Ultramarine Blue 05	Offline a* value	−0.106822

**Table 7:** Pearson correlation coefficients between chemical components and offline b* value.

Chemical Component	Offline Color Data	Pearson Correlation Coefficient
Solvent Yellow 114	Offline b* value	0.3597
PC Makrolon 2807	Offline b* value	0.105443
Plast Red 8355	Offline b* value	0.047905
EBS L-205F	Offline b* value	0.012755
Tiona 288	Offline b* value	−0.059283
Heliogen Green K 8730	Offline b* value	−0.074992
Raven 1010	Offline b* value	−0.102322
Ultramarine Blue 05	Offline b* value	−0.359825

**Table 8:** Pearson correlation coefficients between chemical components and offline L* value.

Chemical Component	Offline Color Data	Pearson Correlation Coefficient
Tiona 288	Offline L*	0.447425
Solvent Yellow 114	Offline L*	−0.008293
PC Makrolon 2807	Offline L*	−0.130144
EBS L-205F	Offline L*	−0.162846
Plast Red 8355	Offline L*	−0.225973
Heliogen Green K 8730	Offline L*	−0.23764
Ultramarine Blue 05	Offline L*	−0.354951
Raven 1010	Offline L*	−0.359394

**Table 9:** Pearson correlation coefficients between inline and offline color data.

Inline Color Data	Offline Color Data	Pearson Correlation Coefficient
Inline L*	Offline L*	0.583606
Inline a*	Offline a*	0.576646
Inline b*	Offline b*	0.522276

**Table 10:** Table of machine learning model and its aggregated dE values.

Model	Aggregated dE
Bagging Regression with Decision Tree Regression	10.84
Deep Neural Networks	22.90
Multiple Linear Regression	25.39
Random Forest	10.75

**Table 11:** Table of comparison of pros and cons between current and previous studies.

Machine Learning Model	Bagging with Decision Tree Regression	Neural Networks	Multiple Linear Regression	Random Forest Regression
Pros	Less probability of overfitting [20]	Incorporated multi-task learning, where learning does not occur solely for one task [11]	Fast calculation speed [14]	Effective for learning with limited samples [26]
	Simple model [20]	Able to implicitly detect complex nonlinear relationships between dependent and independent variables [27]		Robust for learning with strong data error [26]
	Robust to the effect of noisy data [28]			Feasible for nonlinear or approximately linear problems [26]
Cons	Uses significant computational complexity [29]	Unexplained behaviors of the model [30]	Assumes data are normally distributed, homogenous in variance, and independent of one another [31]	Uses significant computational resources, as they require several splitting and evaluations of candidate splits [20]
	Loss of simplicity compared to a simple decision tree [29]	The duration of training a Neural Network is unknown [30]		There are no equations linking the variables with the predicted variable [32]
		Proneness to overfitting [27]		

## Data Availability

Data are contained within the article.

## Appendix A

**Table A1:** Dataset of inline and offline color readings.

Formulation	Inline L*	Inline a*	Inline b*	Offline L*	Offline a*	Offline b*
1	14.37	2.11	6.33	86.68	−0.49	8.87
2	24.74	0.45	5.72	83.06	−0.81	12.15
3	31.44	−0.06	6.43	84.94	−0.17	10.64
4	39.17	0.14	9.71	86.36	0.32	8.24
5	44.48	0.92	13.14	88.01	−0.54	6.13
6	48.25	1.98	16.38	90.95	−0.36	5.56
7	49.93	2.55	18.05	92.94	−0.44	5.23
8	50.57	2.73	18.69	94.06	−0.42	5.02
9	50.94	2.82	19.11	94.69	−0.43	4.58
10	48.46	1.33	12.74	75.55	−0.42	3.21
11	45.14	1.41	11.15	66.74	−0.4	1.29
12	41.25	1.44	9.80	59.63	−0.37	0.29
13	33.54	1.18	6.88	44.66	−0.17	−1.03
14	24.03	1.08	4.19	27.95	0.39	−0.44
15	18.00	1.80	5.10	26.48	0.38	0.07
16	14.40	1.96	5.25	70.81	−56.05	10.94
17	14.55	1.65	5.05	63.96	−68.34	16.37
18	14.73	1.05	4.82	52.7	−70.06	20.14
19	14.86	0.60	4.76	41.81	−53.38	15.94
20	14.96	0.29	4.71	32.2	−26.83	7.7
21	15.07	0.00	4.64	26.06	−5.42	0.62
22	15.14	−0.15	4.59	24.97	−1.18	−0.9
23	15.24	−0.27	4.63	24.87	−0.66	−1.19
24	15.78	1.98	6.40	24.84	1.09	3.15
25	15.88	1.98	6.35	23.97	0.4	1.98
26	15.19	1.96	6.19	23.98	0.4	1.97
27	15.20	1.98	6.24	24.03	0.4	1.94
28	15.26	1.99	6.26	24.03	0.4	1.95
29	15.29	2.01	6.33	24.11	0.4	1.91
30	15.70	2.05	6.53	24.12	0.41	1.94
31	15.44	2.14	6.60	23.96	0.4	2.01
32	16.27	2.13	6.41	81.44	−3.39	1.34
33	16.39	2.09	6.33	76.34	−6.06	−6.03
34	16.50	2.10	6.25	65.04	−10.42	−22.51
35	16.56	2.15	6.12	55.55	−10.49	−36.02
36	16.56	2.19	6.09	42.42	0.09	−50.14
37	16.59	2.21	6.04	34.15	16.07	−54.19
38	16.58	2.24	5.96	30.78	20.03	−50.65
39	16.60	2.29	5.88	27.16	19.01	−40.4
40	16.73	1.94	6.35	82.68	−10.3	79.52
41	17.65	1.19	8.44	82.12	−6.79	85.79
42	18.18	0.47	10.76	79.87	1.66	90.88
43	19.27	−0.59	14.36	76.68	11.56	90.26
44	20.13	−1.29	17.61	73.5	21.74	87.14
45	20.83	−1.69	21.01	70.14	30.64	82.29
46	21.54	−1.47	22.98	67.68	36.11	78.3
47	16.86	2.15	33.52	64.26	42.23	72.51
48	23.20	3.49	21.00	82.69	−5.13	61.86
49	26.16	3.96	35.05	80.77	−1.15	69.22
50	24.52	5.51	40.48	76.42	9.22	76.01
51	22.49	7.12	43.69	72.3	17.24	75.34
52	17.75	2.92	38.77	39.12	−3.05	21.52
53	14.72	1.65	34.56	26	0.69	3.3
54	16.86	2.15	33.52	25.08	0.54	1.71
55	44.78	−0.56	7.72	75.89	−5.33	−6.27
56	43.13	−1.17	5.29	71.97	−6.19	−12.14
57	38.48	−2.03	−0.71	60.6	−5.24	−26.93
58	33.61	−2.12	−6.24	52.94	−1.99	−35.12
59	26.99	−0.62	−0.80	38.43	−2.25	−8.83
60	19.75	1.17	1.70	23.98	0.68	−0.97
61	15.50	1.88	4.61	23.62	0.65	0.53
62	24.01	−7.16	9.50	67.86	−39.25	3.2
63	24.80	−8.09	9.88	62.57	−44.73	8.02
64	22.68	−6.96	9.21	50.62	−46.06	5.83
65	20.43	−5.85	8.68	43	−39.76	6.06
66	16.84	−4.22	8.02	34.84	−18.75	2.12
67	16.12	−2.08	7.01	24.68	−0.58	1.04
68	18.07	1.02	6.25	24.95	−1.37	0.83
69	14.63	3.75	7.24	61.4	63.16	−6.31
70	14.82	4.31	7.53	55.5	71.35	1.46
71	15.03	4.80	7.92	51.42	69.84	17.81
72	15.18	5.22	8.17	48.08	65.4	31.76
73	15.28	5.61	8.39	44.86	61.18	36.52
74	15.40	5.88	8.53	41.3	56.05	33.22
75	15.45	6.02	8.58	39.12	52.5	29.7
76	15.11	6.08	8.48	36.6	47.55	25.41
77	38.76	26.41	5.81	65.73	51.64	3.92
78	35.32	31.16	4.25	61.39	58.12	6.59
79	27.58	33.64	3.26	53.28	62.67	14.54
80	23.15	29.58	4.38	49.94	61.27	17.73
81	19.75	18.42	3.11	34.64	25.15	4.53
82	16.01	7.91	5.62	28.04	10.24	8.2
83	15.10	5.79	6.90	27.16	7.41	7.06
